# The role of water in APCI-MS online monitoring of gaseous *n*-alkanes

**DOI:** 10.1007/s00216-024-05431-5

**Published:** 2024-08-07

**Authors:** Jonas Wentrup, Thomas Dülcks, Jorg Thöming

**Affiliations:** 1 Faculty of Production Engineering, Chemical Process Engineering, University of Bremen, Leobener Strasse 6, 28359 Bremen, Germany; 2 Center for Environmental Research and Sustainable Technology, University of Bremen, Postbox 330 440, 28334 Bremen, Germany; 3 Mass Spectrometry Service Facility, University of Bremen, FB 02Leobener Str. NW2A, 28359 Bremen, Germany; 4 MAPEX Center for Materials and Processes, University of Bremen, Postbox 330 440, 28334 Bremen, Germany

**Keywords:** Gas-phase analysis, APCI mass spectrometry, Hydrocarbons, Humidity, Ionization mechanism, Online monitoring

## Abstract

In atmospheric pressure chemical ionization mass spectrometry (APCI-MS), [M−3H+H_2_O]^+^ ions can deliver analyte-specific signals that enable direct analysis of volatile *n*-alkane mixtures. The underlying ionization mechanisms have been the subject of open debate, and in particular the role of water is insufficiently clarified to allow for reliable process analytics when the humidity level changes over time. This can be a problem, particularly in online monitoring, where analyte accumulation in the ion source can also occur. Here, we investigated the role of water during APCI-MS of volatile *n*-alkanes by changing the carrier gas for sample injection from a dry to a wetted state as well as by using ^18^O-labeled water. This allowed for a distinction between gaseous and surface-adsorbed water molecules. While adsorbed water seems to be responsible for the desired [M−3H+H_2_O]^+^ signals through surface reactions with the analyte molecules, gaseous water was found to promote the formation of C_*n*_H_2*n*+1_O^+^ of different (and analyte-independent) hydrocarbons, revealing a reaction with hydrocarbon species which accumulated in the ion source during continuous operation. At the same time, gaseous water competed with analyte molecules for ionization and thus suppressed the formation of alkyl (C_*n*_H_2*n*+1_^+^) and alkenyl (C_*n*_H_2*n*−1_^+^) ions. The results reveal a memory effect due to hydrocarbon adsorption, which may cause severe interpretation difficulties when the ionization chamber undergoes sudden humidity changes. The use of [M−3H+H_2_O]^+^ for *n*-alkane analysis in alkane/water mixtures can be facilitated by constantly maintaining high humidity and hence stabilizing the ionization conditions.

**Graphical abstract:**

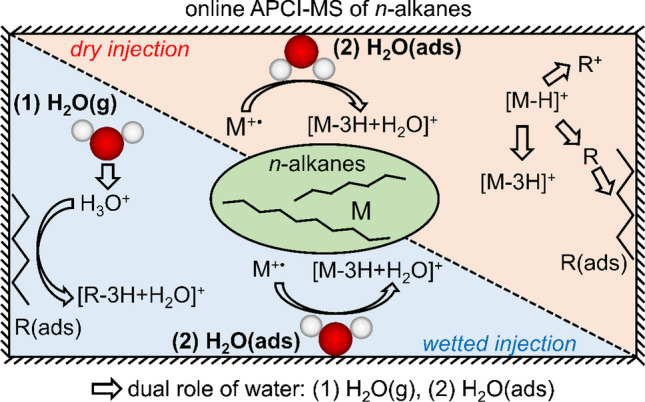

**Supplementary Information:**

The online version contains supplementary material available at 10.1007/s00216-024-05431-5.

## Introduction

Atmospheric pressure chemical ionization mass spectrometry (APCI-MS) can be used for analyzing hydrocarbon analytes over a range from volatile organic compounds (VOCs) [1–3] up to base oils and heavy petroleum products [4–8]. When measuring saturated hydrocarbons, most APCI studies focus on the detection of carbenium ions ([M−H]^+^), which are usually highly abundant [6, 7, 9–12]. Various reaction routes for [M−H]^+^ formation have been postulated in the literature. The most prominent example implies a hydride abstraction mechanism [1, 9, 12–14] (e.g., by Eqs. 1 and 2). However, Manheim et al. specifically ruled out such a pathway in their study and determined a proton transfer by H_3_O^+^ and N_2_H^+^ with subsequent H_2_ elimination (Eqs. 3 and 4) to be mainly responsible for [M−H]^+^ formation [15].

1 $$\text{M }+{\text{NO}}^{+}\to {\left[\text{M}-\text{H}\right]}^{+}+\text{HNO},$$

2 $$\text{M }+{\text{R}}^{+}\to {\left[\text{M}-\text{H}\right]}^{+}+\text{RH},$$

3 $$\text{M}+{\text{H}}_{3}{\text{O}}^{+}\to {\left[\text{M}-\text{H}\right]}^{+}+{\text{H}}_{2}\text{O}+{\text{H}}_{2},$$

4 $$\text{M}+{\text{N}}_{2}{\text{H}}^{+}\to {\left[\text{M}-\text{H}\right]}^{+}+{\text{N}}_{2}+{\text{H}}_{2}.$$

Moreover, although APCI is characterized as a “soft” ionization technique, [M−H]^+^ ions are known to undergo further fragmentation, leading to, among others, [M−3H]^+^ by successive H_2_ elimination (Eq. 5) as well as to smaller alkyl and alkenyl ions by carbon bond cleavage (Eqs. 6 and 7) [1, 3, 15].

5 $${[\text{M}-\text{H}]}^{+} \to {[\text{M}-3\text{H}]}^{+}+{\text{H}}_{2},$$

6 $${[\text{M}-\text{H}]}^{+} \to {{\text{R}}_{\text{alkyl}}}^{+}+\text{neutral fragment},$$

7 $${[\text{M}-3\text{H}]}^{+} \to {{\text{R}}_{\text{alkenyl}}}^{+}+\text{neutral fragment}.$$

The overlap of identical [M−H]^+^ fragment ions for different *n*-alkanes complicates an analysis of mixtures [8]. In our previous work, we showed that oxygen-containing [M−3H+H_2_O]^+^ ions can be beneficial for quantifying volatile and semi-volatile (~C_5_–C_20_) *n*-alkanes in gaseous streams, since they produce analyte-specific signals [16]. Hardly any fragments with the molecular formula C_*n*_H_2*n*+1_O^+^ were observed, which allowed for an easy and reproducible [M−3H+H_2_O]^+^ signal allocation, even in mixtures of multiple *n*-alkanes. Still, it remained an open question how [M−3H+H_2_O]^+^ were formed and why no overlapping fragment ions were observed. Moreover, especially for larger alkanes, long-tailing ion signals were detected, indicating an analyte accumulation in the ion source. In a time-optimized online sample injection application without cleaning procedures in between, this continuous analyte accumulation in the ion source may lead to an interference of the related signals with actual sample signals.

The formulation [M−3H+H_2_O]^+^ assumes an ion formation with water, and several researchers agree on a hydration mechanism [3, 6, 9, 14], e.g., by

8 $${\left[\text{M}-3\text{H}\right]}^{+}+{\text{H}}_{2}\text{O}\to {\left[\text{M}-3\text{H}+{\text{H}}_{2}\text{O}\right]}^{+},$$

but an oxidized [M−H+O]^+^ molecule is also imaginable. Nyadong et al. confirmed water as oxygen source by adding ^18^O-labeled water to the system [6]. However, other researchers ruled out water as oxygen source and determined a reaction with ozone (O_3_), produced by O_2_ during corona discharge, as the mechanism responsible for the formation of [M−H+O]^+^ [17]. Evidently, APCI ionization routes of saturated hydrocarbons are still under debate and may even differ, depending on applied ionization conditions.

Water is generally known to be omnipresent during APCI-MS, since air humidity is usually inevitable. But in most literature studies, the role of water is limited to the source of H_3_O^+^ ions and water clusters [18–20], which may act as proton donors in subsequent ionization steps (as, e.g., in Eq. 3). Oxygen-containing ions, such as [M−3H+H_2_O]^+^/[M−H+O]^+^, are typically mentioned as a side issue only. However, a clear understanding of the processes leading to their formation is crucial when these ions are to be used for hydrocarbon quantification, especially in applications where water is produced along with hydrocarbons (e.g., biomass pyrolysis [21] or Fischer–Tropsch synthesis [22]).

In this study, we investigate the role of water during online APCI(+)-MS of gaseous *n*-alkanes. In this context, we particularly differentiate between two water states, i.e., gaseous water and water adsorbed to surfaces within the ion source. The results lead to the proposition of an ionization scheme for the formation of [M−H]^+^, [M−3H]^+^ ions, their fragments and of [M−3H+H_2_O]^+^ ions. Moreover, the problem of hydrocarbon accumulation in the ion source and the accompanying influence of water is outlined. We discuss the meaning of the evolved APCI mechanism for an online analysis application of *n*-alkane/water mixtures and provide a first approach for system stabilization.

## Materials and methods

### Reagents

*n*-Pentane (Sigma-Aldrich, ≥ 99% purity), *n*-hexane (VWR chemicals, ≥ 95% purity), *n*-heptane (VWR chemicals, 99.9% purity), *n*-decane (Thermo Scientific, 99% purity), *n*-dodecane (VWR chemicals, ≥ 99% purity) and *n*-tetradecane (Alfa Aesar, 99% purity) were used as alkane standards. ^18^O-water (97% purity) was purchased from ABX Advanced Biochemical Compounds (Radeberg, Germany). Conventional ^16^O-water was taken from the in-house deionized water tap.

### Experimental setup

**Fig. 1:**
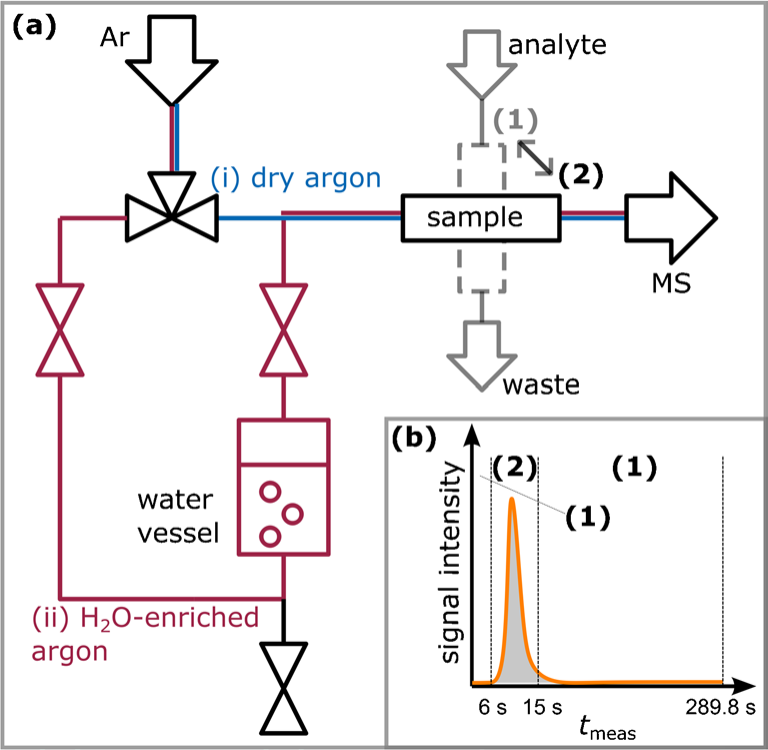
MS sample injection configuration. **a** Argon carrier gas usage with either (i) dry argon conditions or (ii) water-enriched argon. Additionally, the sample valve positions for (1) sampling and (2) injection are shown. **b** A typical plot of signal intensity over measurement time during one sample injection and the corresponding valve positions (1) and (2)

Gaseous samples of diluted *n*-alkanes were analyzed using a Q Exactive Plus Orbitrap mass spectrometer (Thermo Fisher, Waltham, MA, USA) and an 8860 gas chromatograph (Agilent, Santa Clara, CA, USA) for reference. The hydrocarbons were injected into an evaporation chamber via a syringe pump and transported by diluted syngas mixture (CO/H_2_/N_2_ = 1:2:1) to both instruments. Argon was used as carrier gas to flush the sample into the APCI ion source. In order to add water to the ionization chamber, a water-filled tube (~ 5 ml capacity) was inserted into the argon carrier gas pipeline, so that the gas stream could be enriched with water before it flushed the sample into the MS. A three-way valve allowed for switching between dry argon carrier gas and water-enriched argon carrier gas (Fig. 1a). The MS sample injection was performed automatically between 6 s ≤ *t*_meas_ < 15 s, and the overall measurement duration was about 5 min (289.8 s). An illustrative plot of signal intensity over time during one sample measurement is shown in Fig. 1b. A signal integration over time results in a peak area, which is used for a correlation with the analyte concentration. More details about the experimental setup, the APCI-MS method, signal reproducibility as well as about the reference GC measurements, can be found in our previous study [16].

### Effect of gaseous water

A C_5_/C_6_/C_7_/C_10_/C_12_/C_14_ (mole fractions *y*_*i*_ = 0.403/0.282/0.198/0.068/0.033/0.016) *n*-alkane mixture was injected by a syringe pump into the evaporation zone with a constant flow rate of 6 µl/min. The hydrocarbons were transported by a diluted syngas mixture (CO/H_2_/N_2_ = 1:2:1, 200 ml_STP_/min) to both the MS instrument and a reference gas chromatograph. The MS argon carrier gas flow was started in dry argon mode. After reaching a steady state, the argon was switched to the water bath filled with ^18^O-water. This way, the effect of gaseous water molecules could be distinguished from water molecules that were present in dry argon mode.

### Direct H_2_^18^O injection

Before performing another alkane experiment, a H_2_^18^O sample of 600 µl was directly injected into the ionization chamber for 3 min. This aimed to study the effect of incoming water on possible hydrocarbon accumulation and further to enrich the ionization chamber with H_2_^18^O molecules, leading to partial H_2_^18^O condensation and surface accumulation. To enhance this process, the vaporizer temperature ${T}_{\text{vap}}$ was lowered to 100 °C. Mass spectra were continuously measured during the injection and the time afterwards to monitor possible effects of the strong humidity increase.

### Effect of adsorbed water

After the direct H_2_^18^O injection, the ion source temperature was brought back to normal operation conditions for a C_10_ injection experiment. *n*-decane was injected with a constant flow rate of 1 µl/min into a N_2_ gas stream (50 ml_STP_/min), evaporated and guided to the MS device. It had been found that, using the previous MS sheath gas conditions from the sections “Effect of gaseous water” and “Direct H_2_^18^O injection” (sheath gas: 2, sweep gas: 10), ionization efficiency was rather low, so that small amounts of ^18^O-species were hardly detectable. Thus, in this experiment, the MS sheath gas flow was increased to 40 and the sweep gas was turned off, which enhanced signal intensities and decreased the lower detection limit. Similar to the other experiment, the argon carrier gas was dry at first, then switched to the water bath filled with ^18^O-water and, after reaching a new steady state, switched back to dry argon mode.

## Results and discussion

### Effect of gaseous water

**Fig. 2:**
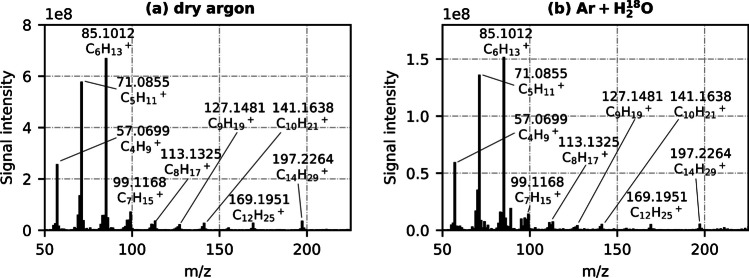
Exemplary mass spectra of a C_5_/C_6_/C_7_/C_10_/C_12_/C_14_ sample injection using **a** dry argon as carrier gas and (**b**) H_2_^18^O-enriched argon carrier gas

Two exemplary mass spectra of the C_5_/C_6_/C_7_/C_10_/C_12_/C_14_ mixture, showing one MS scan during a measurement with dry argon carrier gas flow and one MS scan during a measurement with H_2_^18^O-wetted argon flow, are presented in Fig. 2a and b, respectively.

Both scans correspond to an equal point in time during their measurement (*t*_meas_ = 10.8 s). The spectra look qualitatively similar: Most abundant species are alkyl ions (C_*n*_H_2*n*+1_^+^), especially with chain lengths *n* = 4, 5 and 6 (*m/z* 57.0699, *m/z* 71.0855, and *m/z* 85.1012, respectively). This is in agreement with our previous study, and highlights the difficulty in quantifying alkane mixtures using [M−H]^+^, since the degree of fragmentation is high and hence the attribution of peaks to different analytes is infeasible. Here, for instance, it is impossible to distinguish between *m/z* 71.0855 (C_5_H_11_^+^) originating from *n*-pentane or from a fragmentation of one of the higher *n*-alkanes. Quantitatively, the additional presence of water molecules leads to a significant decrease in signal intensity (see Fig. 2b). This decrease was observed regardless of an argon enrichment with H_2_^18^O or conventional H_2_^16^O (not shown here). Evidently, the addition of gaseous water inhibits the formation of [M−H]^+^ ions and their fragments. Ions, which would suggest a reaction of alkyl ions with water to other species, were not observed. Hence, it seems that water molecules competed with analyte molecules and were preferably ionized. Main ionization products were presumably H_3_O^+^ and water clusters with *m/z* < 50, which could not be detected. This indicates that under the applied ionization conditions, [M−H]^+^ and fragment formation is not due to proton transfer reactions (Eqs. 3 and 4), since a higher availability of H_3_O^+^ ions would suggest a signal increase rather than a decrease. Instead, another mechanism, e.g., hydride abstraction, could be predominant. This is contradictory to the results of Manheim et al., who ruled out hydride abstraction and determined a proton transfer mechanism to be dominant [15]. Still, their mechanistic study was performed under vacuum and lower temperature conditions, which could explain different reaction regimes.

**Fig. 3:**
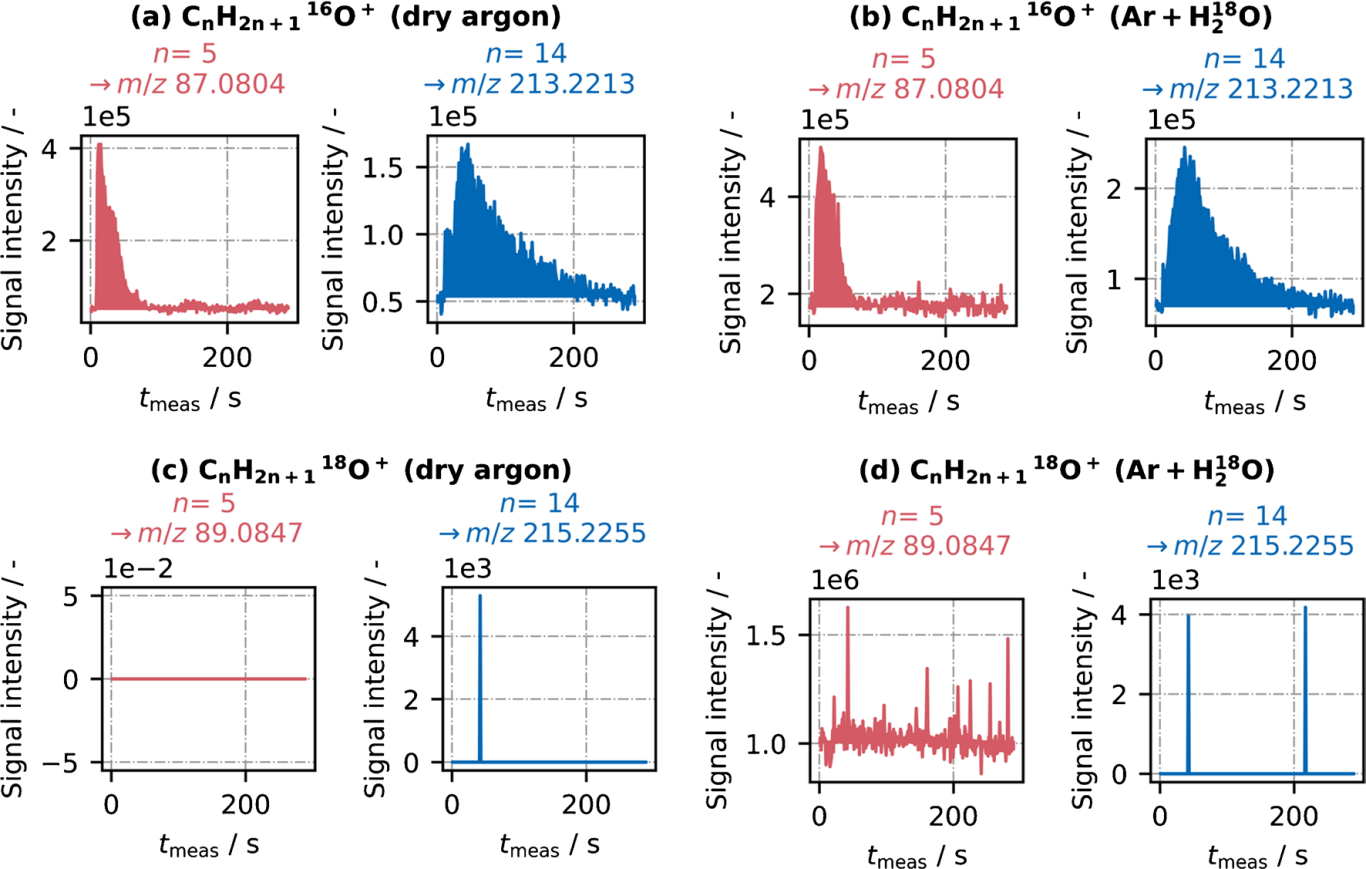
MS ion chromatograms of oxygen-containing C_*n*_H_2*n*+1_^16^O^+^ (**a** and **b**) and C_*n*_H_2*n*+1_^18^O^+^ (**c** and **d**) with the chain lengths *n* = 5 and *n* = 14, using dry argon carrier gas and H_2_^18^O-enriched argon carrier gas, respectively

With respect to oxygen-containing C_*n*_H_2*n*+1_O^+^ ions, the influence of gaseous water is more complex. Figure 3 presents the signals of C_*n*_H_2*n*+1_O^+^ during one C_5_/C_6_/C_7_/C_10_/C_12_/C_14_ sample injection using dry argon as well as during one C_5_/C_6_/C_7_/C_10_/C_12_/C_14_ sample injection using H_2_^18^O-wetted argon. For the sake of simplicity, only the signals of the smallest (*n* = 5; red) and the largest ion species (*n* = 14; blue) are shown. The signals of the other four analytes (as well as for the corresponding alkyl and alkenyl ions) are shown in Figs. S1 and S2 in the Supporting Information. We specifically distinguish between C_*n*_H_2*n*+1_^16^O^+^ (panels a, b) and C_*n*_H_2*n*+1_^18^O^+^ (panel c, d), to check the influence of the incoming H_2_^18^O. Generally, C_*n*_H_2*n*+1_O^+^ ion abundance was significantly lower than that of alkyl ions (magnitude of 10^5^–10^6^ vs. 10^7^–10^8^).

In dry argon mode, clear signals were observed for C_*n*_H_2*n*+1_^16^O^+^ ions, which means that their formation is based on either intrinsic humidity or a different oxygen source such as O_2_ or O_3_. No signals were observed for C_*n*_H_2*n*+1_^18^O^+^ ions with dry argon, which is reasonable, since H_2_^18^O had not been added yet and the ^16^O/^18^O ratio in natural water is only about 0.2% [23]. When the argon carrier gas was enriched with H_2_^18^O, both isotopic ion groups increased in signal abundance, but in a different manner: C_*n*_H_2*n*+1_^16^O^+^ peak areas increased, most significantly for the longer analytes (~5% increase for C_5_H_11_^16^O^+^ vs. ~60% increase for C_14_H_29_^16^O^+^). In contrast, C_14_H_29_^18^O^+^ still showed no relevant signal, while ions with a shorter chain length, such as C_5_H_11_^18^O^+^, were detected with high abundance (magnitude of 10^6^). In addition, it is striking that the signal development of C_5_H_11_^18^O^+^ did not show a typical peak shape. Instead, the ions were continuously detected over the entire measurement period, i.e., even before the current sample injection. Hence, the added gaseous water interacted with hydrocarbon molecules, but obviously not with the ones of the current sample. The constantly detected C_5_H_11_^18^O^+^ signal must originate at least partly from accumulated hydrocarbons (from previous injections), and the “new” sample molecules (such as C_14_) seem to be unaffected. This leads to two conclusions: First, hydrocarbon accumulation is present and becomes apparent when adding gaseous water. Incoming water seems to react with accumulated hydrocarbons, which are then remobilized and detected. This issue will be further discussed in the section “Direct H_2_^18^O injection.” Second, analyte-specific [M−3H+H_2_O]^+^/[M−H+O]^+^ ions were obviously not formed by reactions with gaseous water. If this were the case, C_*n*_H_2*n*+1_^18^O^+^ should have been detected for all analytes. Nevertheless, an increase in the peak areas of C_*n*_H_2*n*+1_^16^O^+^ was observed. Hence, the addition of gaseous water seems to enhance [M−3H+H_2_^16^O]^+^ formation via an indirect reaction pathway, i.e., by supporting an analyte interaction with a different oxygen source. Two possible oxygen-containing reactants that are promoted by the presence of gaseous water are imaginable: (1) ions which are based on atmospheric O_2_ and N_2_ and which form hydrates with gaseous water, e.g., ${{\text{O}}_{2}}^{+\bullet }{({\text{H}}_{2}\text{O})}_{n}$ or ${\text{NO}}^{+\bullet }{({\text{H}}_{2}\text{O})}_{n}$ [1, 24]; (2) adsorbed water, which is predominantly H_2_^16^O(ads), and increases in reactivity due to higher water partial pressure in the ion source (similar to heterogeneously catalyzed reaction rates). Although hydrates such as ${{\text{O}}_{2}}^{+\bullet }{\left({\text{H}}_{2}\text{O}\right)}_{n}$ or ${\text{NO}}^{+\bullet }{\left({\text{H}}_{2}\text{O}\right)}_{n}$ might be formed during the ionization process, they were not detected here (*n* = 1 could not be detected anyway due to *m/z* < 50), and moreover, a transfer of only one oxygen atom from those ions to form [M−H+O]^+^ is unrealistic. Hydrocarbon adducts with O_2_^+^ or NO^+^ seem more reasonable. Thus, the second route, i.e., an increased probability of primary hydrocarbon ion reactions with adsorbed water molecules forming [M−3H+H_2_O]^+^ due to higher water partial pressure, is more plausible, especially since this also explains a prolonged signal detection due to slow wall interactions. However, this scenario still needs to be confirmed.

### Direct H_2_^18^O injection

**Fig. 4:**
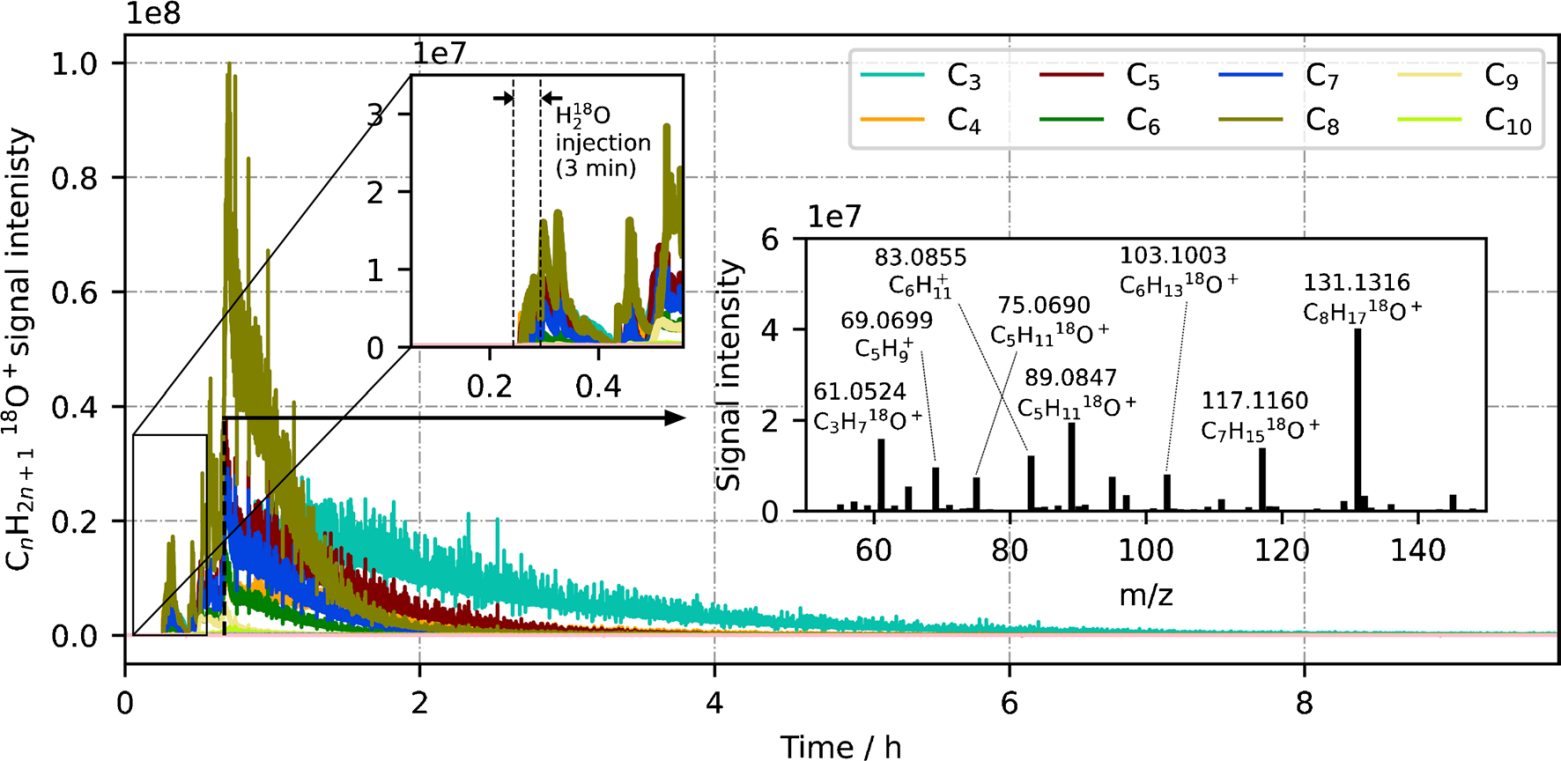
Temporal development of C_*n*_H_2*n*+1_^18^O^+^ signals during and after H_2_^18^O injection

The observed reaction of water with accumulated hydrocarbons was further examined by a pure and direct H_2_^18^O injection with no additional hydrocarbon analytes involved. Figure 4 shows the signal development of C_*n*_H_2*n*+1_^18^O^+^ before, during, and after H_2_^18^O injection. The results corroborate the issue of hydrocarbon accumulation in the ion source. Although only ^18^O-water was injected in this experiment, i.e. no hydrocarbons were added, a high amount of C_*n*_H_2*n*+1_^18^O^+^ ions in the range of *n* = 3–10 were measured. Hence, there has been a significant accumulation of hydrocarbon molecules which were remobilized by the added water. This reinforces the observation of the previous experiment that gaseous water (presumably via H_3_O^+^) reacts with adsorbed hydrocarbon molecules that did not originate from a current sample injection, but from former sample injections (Eq. 9). Moreover, since for instance *n*-propane (C_3_), *n*-butane (C_4_), and *n*-octane (C_8_) were never used as analytes before, a high fraction of adsorbed species evidently consists of adsorbed (and presumably neutral) fragments.

Remarkably, this ion formation was visible not only during the 3 min of H_2_^18^O injection, but for several hours afterwards as well. Interestingly, smaller molecules were detected for a longer period than larger ones, since C_3_H_7_^18^O^+^ in particular was found to be a very persistent signal. This might be due to a higher relative polarity of short-chained fragments, which causes stronger interactions with the hydrophilic walls.

The inset in Fig. 3 shows an exemplary mass spectrum captured during this process. The C_*n*_H_2*n*+1_^18^O^+^ signals were the most abundant, highlighting that this type of oxygen-containing species is a particularly stable reaction product. The alkenyl cations C_5_H_9_^+^ (*m/z* 69.0699) and C_6_H_11_^+^ (*m/z* 83.0855) were also very abundant, which suggests that they were formed via a water-based pathway as well, but with subsequent water elimination (Eq. 10). We propose the following ionization mechanism for gaseous H_3_O^+^ ions with adsorbed neutral, saturated hydrocarbon molecules:

9 $${{\text{H}}_{3}\text{O}}^{+}\left(\text{g}\right)+{\text{C}}_{n}{\text{H}}_{2n+2}\left(\text{ads}\right)\to {\left[{\text{C}}_{n}{\text{H}}_{2n+2}-3\text{H}+{\text{H}}_{2}\text{O}\right]}^{+}\left(\text{g}\right)+2{\text{H}}_{2}\left(\text{g}\right),$$

10 $${\left[{\text{C}}_{n}{\text{H}}_{2n+2}-3\text{H}+{\text{H}}_{2}\text{O}\right]}^{+}\left(\text{g}\right)\to {\left[{\text{C}}_{n}{\text{H}}_{2n+2}-3\text{H}\right]}^{+}\left(\text{g}\right)+{\text{H}}_{2}\text{O}\left(\text{g}\right).$$

When using APCI-MS as a continuous online monitoring method, this means of course a problematic situation, since signals from analytes and accumulated species overlap, and no reliable quantification can be made. However, we did not observe this phenomenon during our last study, where only *n*-alkanes (no water) were investigated. Hence, this problem seems to be significant only when water is added. This is an interesting mechanistic observation, as it means that nonpolar accumulated hydrocarbons may be effectively cleaned by polar water instead of nonpolar solvents due to remobilizing surface reactions.

### Effect of adsorbed water

**Fig. 5:**
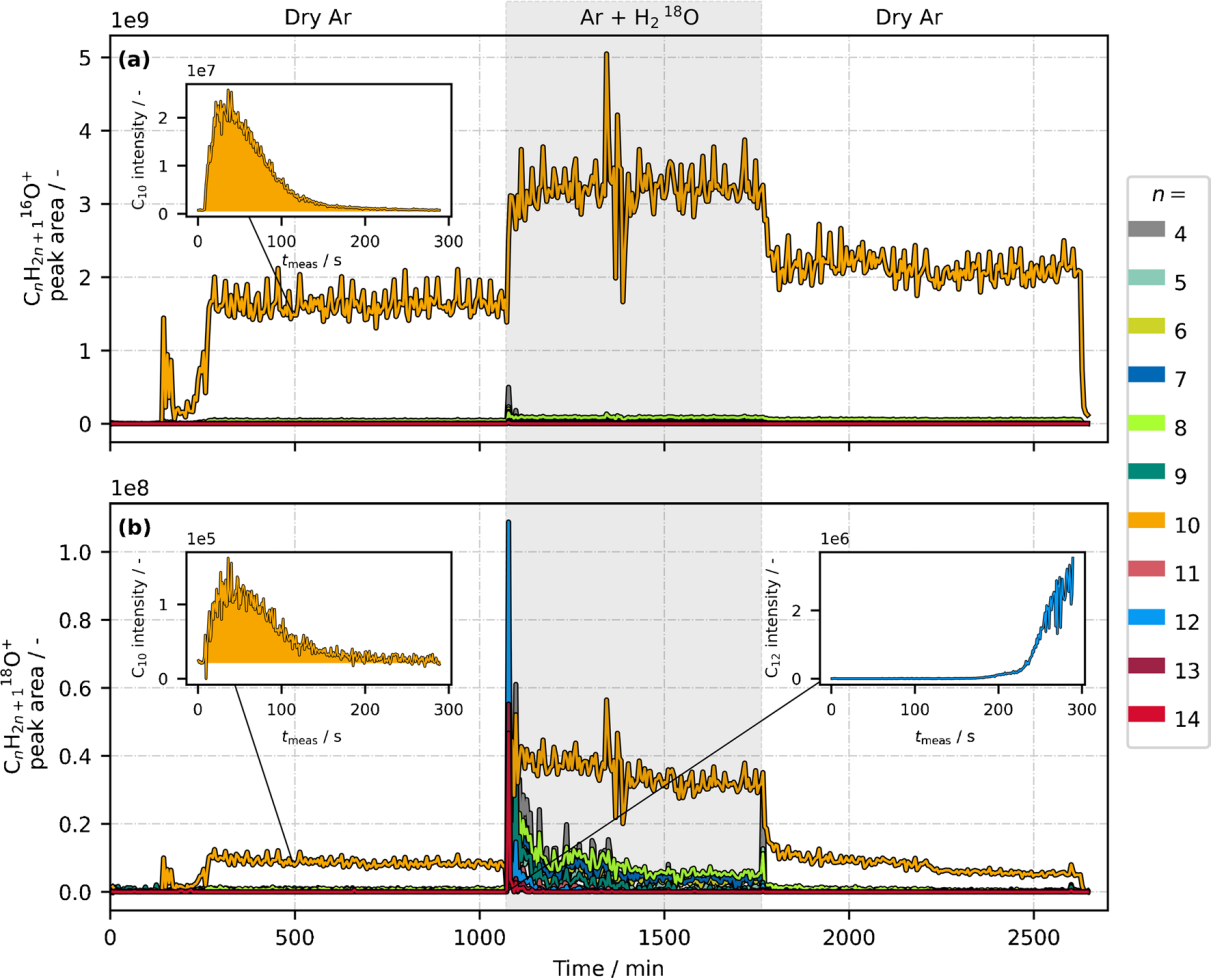
Peak areas of the ion groups **a** C_*n*_H_2*n*+1_^16^O^+^, **b** C_*n*_H_2*n*+1_^18^O^+^during a constant *n*-decane injection and a switch between dry (white background) and H_2_^18^O-wetted (shaded background) argon carrier gas. The insets show the signal curve of the particular ion at the marked time, respectively

After the direct injection of H_2_^18^O, we expected that the surface inside the ionization chamber would be at least partly covered with ^18^O-labeled water molecules. This would allow us to check whether they interact with the analyte *n*-decane. Furthermore, the effect of additional gaseous H_2_^18^O could be monitored by switching between dry and wetted argon mode. Figure 5a and b show the temporal development of peak areas for the ion groups C_*n*_H_2*n*+1_^16^O^+^ and C_*n*_H_2*n*+1_^18^O^+^, respectively. Not only is *n* = 10 presented, but the chain lengths *n* = 4–14 are also shown. This way, the fragmentation patterns of C_*n*_H_2*n*+1_^16^O^+^ can be compared with C_*n*_H_2*n*+1_^18^O^+^, as well as possible larger accumulated molecules which might be released by gaseous water. The inhibiting effect of water on alkyl and alkenyl cations during this experiment is shown in Fig. S3 in the Supporting Information.

Up to a time of ~1070 min, dry argon was used as carrier gas (white background). Both [M−3H+H_2_^16^O]^+^ and [M−3H+H_2_^18^O]^+^ (i.e., C_*n*_H_2*n*+1_O^+^ with *n* = 10) were detected, while C_*n*_H_2*n*+1_^16^O^+^ and C_*n*_H_2*n*+1_^18^O^+^ fragments were hardly visible. Hence, this time, the chemical behavior of both C_*n*_H_2*n*+1_O^+^ isotopes seems to be identical. Moreover, both [M−3H+H_2_^16^O]^+^ and [M−3H+H_2_^18^O]^+^ signals were detected with similar temporal behavior (insets in Fig. 5a and b at t ≈ 500 min). This indicates that the partial coverage of ion source surfaces with H_2_^18^O was successful, and the formation pathway of [M−3H+H_2_O]^+^ via adsorbed water was a valid assumption. No gaseous water was added up to this point, i.e., these ions probably originated from water molecules which were adsorbed to the ion source walls. It is still striking that, despite the previous H_2_^18^O injection, the signal of [M−3H+H_2_^16^O]^+^ is about two orders of magnitude higher than [M−3H+H_2_^18^O]^+^. The peak area fraction of [M−3H+H_2_^18^O]^+^ with respect to all [M−3H+H_2_O]^+^ signals was about 0.51% during the first dry argon period. Apparently, ^16^O-water was still predominantly available on the surfaces. It is likely that greater effort to exclude atmospheric humidity would be needed to replace H_2_^16^O(ads) by H_2_^18^O(ads) to a higher degree. Metal surfaces are known be covered with water [25, 26], and the continuous exposure to atmospheric conditions still allows H_2_^16^O to preferably occupy open surface sites.

The question arises as to which ions react with surface-adsorbed water to form [M−3H+H_2_O]^+^. Different scenarios are imaginable, but since no fragments of C_*n*_H_2*n*+1_O^+^ were observed, a reaction of early-formed (and thus not yet fragmented) analyte ions seems probable. If [M−H]^+^ or [M−3H]^+^ would react with adsorbed water, we would expect their fragments to behave analogously, and the C_*n*_H_2*n*+1_O^+^ fragmentation pattern would look similar to that of the other ion types. Instead, we suggest a reaction mechanism based primarily on formed molecular radical cations:

11 $$\text{M}\left(\text{g}\right)+{{\text{N}}_{2}}^{+\bullet }\left(\text{g}\right)\to {\text{M}}^{+\bullet }\left(\text{g}\right)+{\text{N}}_{2}\left(\text{g}\right),$$

12 $${2\text{M}}^{+\bullet }\left(\text{g}\right)+{2\text{H}}_{2}\text{O}\left(\text{ads}\right)\to {2\left[\text{M}-3\text{H}+{\text{H}}_{2}\text{O}\right]}^{+}\left(\text{g}\right)+3{\text{H}}_{2}.$$

This cannot be verified, and both intermediate reaction steps and more involved species are imaginable. Still, this reaction could explain the low degree of fragmentation of C_*n*_H_2*n*+1_O^+^ ions: ${\text{M}}^{+\bullet }$ ions are formed in a first step and hold a large amount of internal energy. By colliding with the ion source walls and reacting with adsorbed water, a part of that internal energy is transferred to the surface. Desorbing products possess significantly less internal energy and reach the detector without major fragmentation. When the argon stream was guided through the ^18^O-water bath (*t* ~1070 min), both water-based mechanisms became visible: (1) The reaction of analyte ions with surface-adsorbed water (Eq. 12) became more probable due to higher water partial pressure. Because the surface was covered with both H_2_^16^O and H_2_^18^O, the abundance of both [M−3H+H_2_^16^O]^+^ and [M−3H+H_2_^18^O]^+^ strongly increased. (2) Incoming gaseous water reacted with adsorbed hydrocarbons (Eq. 9). Various C_*n*_H_2*n*+1_^18^O^+^ species were immediately detected after adding H_2_^18^O, including chain lengths of *n* > 10, which cannot be fragments of *n*-decane but must originate from previous experiments. With continuous ^18^O-water addition, their signal abundance again decreased. Thus, a part of the analyte signal [M−3H+H_2_^18^O]^+^ presumably originates from previous samples and not from ionized analytes, since the signal first increased strongly and declined subsequently.

The instantaneous release of adsorbed hydrocarbons is further demonstrated in the inset signal curve of C_12_H_25_^18^O^+^ (blue) in Fig. 5b. It represents the moment where argon was switched from dry to ^18^O-wetted mode (at about half time of the measurement). The C_12_ signal increased immediately after that switching event, although sample injection already occurred minutes ago.

When the argon stream was brought back to dry conditions (*t* ~1750 min), [M−3H+H_2_^16^O]^+^ and [M−3H+H_2_^18^O]^+^ signals decreased again, showing the effect of decreasing water partial pressure. A memory effect was observed, as peak areas decreased rather slowly compared to the steep increase after the first switching event. This further supports the scenario of a surface-related reaction scheme, in which a water-saturated state should decline rather slowly. Hence, adsorbed water molecules would remain available for a long period. One could expect this behavior especially for [M−3H+H_2_^18^O]^+^, since H_2_^18^O was added for several hours. However, the signal of [M−3H+H_2_^18^O]^+^ decreased slowly but constantly (even below the level of the first dry argon mode), while the peak areas of [M−3H+H_2_^16^O]^+^ reached a steady state with a slightly higher abundance compared to the first dry argon mode. This could further emphasize that replacing H_2_^16^O(ads) by H_2_^18^O(ads) was rather unlikely, especially when only small amounts of H_2_^18^O where added via argon. The available amount of adsorbed H_2_^18^O molecules appears to originate primarily from the previous direct (liquid) H_2_^18^O injection and hence decreased constantly afterwards. In contrast, H_2_^16^O(ads) was still excessively available.

### Proposal of an ionization scheme

**Fig. 6:**
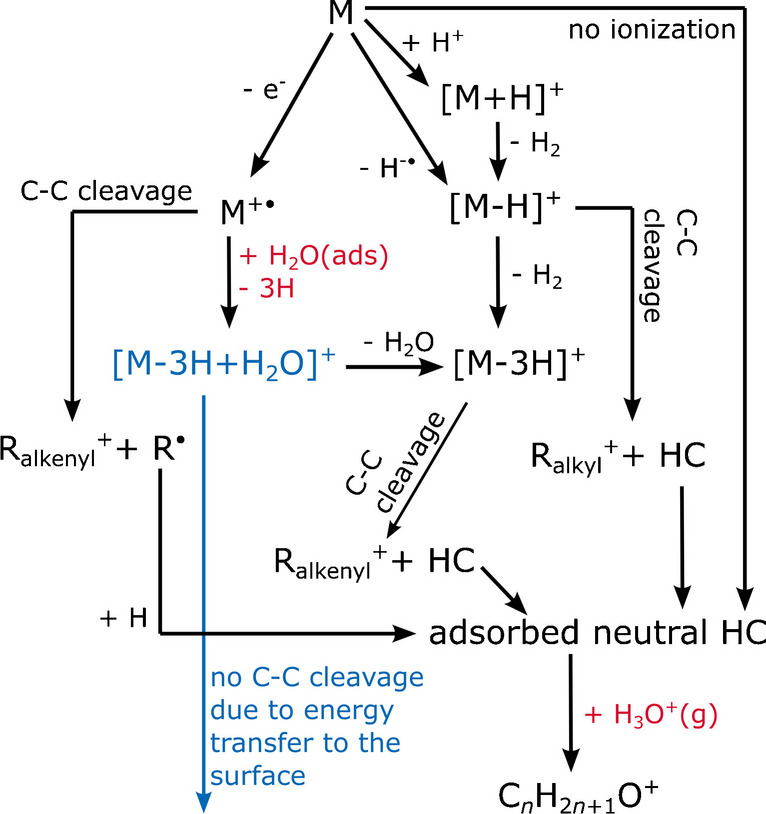
Proposed ionization scheme during APCI-MS analysis of volatile *n*-alkanes. The dual role of water is highlighted in red: adsorbed water (H_2_O(ads)) leads to the formation of [M−3H+H_2_O]^+^ ions, which hardly suffer from further fragmentation (blue pathway). Gaseous water reacts in the form of H_3_O^+^(g) with adsorbed neutral hydrocarbons (former analyte or fragment molecules)

Based on all experimental results as well as on previously described APCI ionization mechanisms, we propose a general scheme for the formation of M^+•^, [M−H]^+^, [M−3H]^+^, [M−3H+H_2_O]^+^ ions and hydrocarbon fragments according to Fig. 6. The formation of [M−H]^+^ ions and other alkyl ions decreased upon addition of water, although presumably more H_3_O^+^ ions were available as proton donators. Hence, a predominant reaction route via proton transfer, as postulated by Manheim et al. [15], is questionable here. Instead, under the applied conditions, [M−H]^+^ ions might be formed by hydride abstraction (Eqs. 1 and 2). Since this was not investigated further, both pathways are added to the scheme. [M−3H]^+^ is formed by a subsequent H_2_ elimination (Eq. 5). Both ion types undergo significant chain fragmentation (Eqs. 6 and 7). [M−3H+H_2_O]^+^ ions (highlighted in blue) are suggested to be formed by a reaction of molecular radical ions with adsorbed water (Eq. 12) and, due to an energy transfer to the wall, they do not suffer from major chain cleavage. Subsequent water elimination is possible, which enables an alternative route for [M−3H]^+^ formation. Any neutral hydrocarbon species (analyte or fragment) can adsorb to the surface and is thus available to participate in the ionization process of subsequent samples. This accumulation or memory effect becomes visible when gaseous water is added, forming H_3_O^+^ ions which remobilize adsorbed hydrocarbons and react to C_*n*_H_2*n*+1_O^+^ ions (Eq. 7).

These mechanistic considerations cannot be entirely proven, but they provide a suitable model for how water is involved during this APCI-MS study of *n*-alkanes. With respect to an online MS application, two important conclusions can be drawn from these results. First, regardless of whether [M−H]^+^, [M−3H+H_2_O]^+^, or other ions are used for quantification, the available amount of water within the ion source significantly affects probable ionization routes. Hence, *n*-alkanes cannot be characterized in a reliable way when the water concentration changes at the same time. Second, without proper cleaning procedures, hydrocarbon accumulation in the ion source may be severe. As long as no water is added, this accumulation remains rather unnoticed. However, additional water remobilizes these species in terms of C_*n*_H_2*n*+1_O^+^ ions, which overlap with signals of the current sample. This leads to a significant interpretation problem when [M−3H+H_2_O]^+^ ions are desired for *n*-alkane analysis, and the humidity level changes over time. At the same time, this means that water might be a suitable cleaning agent for accumulated hydrocarbons.

For reaction processes, where in addition to hydrocarbons, water is formed as a product (e.g., Fischer–Tropsch synthesis), we therefore suggest that the ion source be constantly enriched with water. This way, hydrocarbon accumulation should be reduced, and moreover, predominant ionization mechanisms are kept constant, i.e., no drastic signal changes can be expected when additional water is present within the product sample. An application to Fischer–Tropsch synthesis in currently in progress. A first proof of principle is given in the next section.

### Measuring alkane/water mixtures under saturated humidity conditions

We performed another online monitoring experiment of *n*-decane and constantly moistened the argon carrier gas stream, this time with conventional H_2_^16^O. The MS nitrogen supply was reset to the standard conditions of our previous study (sheath gas: 2; sweep gas: 10) [16]. Similarly, in addition to N_2_, the same diluted syngas mixture (CO/H_2_/N_2_ = 1:2:1, 200 ml_STP_/min) was applied as gas-phase matrix. Again, *n*-decane was injected with 1 µl/min, evaporated, and transported to the MS and GC instrument. A second syringe pump was installed to the evaporation zone of the setup, adding water (H_2_^16^O) to the gas stream. Different water volume flows (1; 10; 30 µl/min) were applied to check the effect of varying water concentrations.

**Fig. 7:**
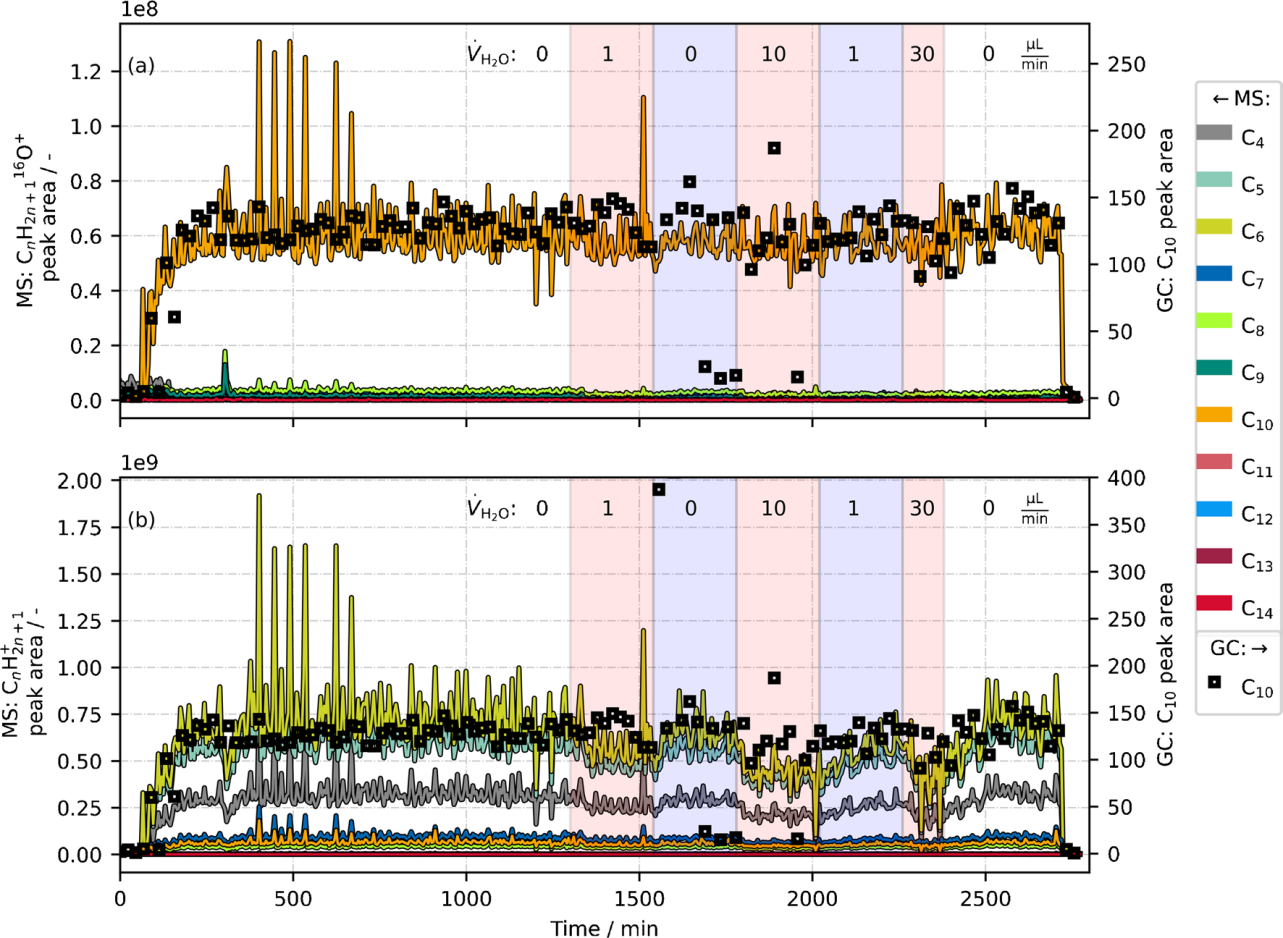
MS peak areas of **a** C_*n*_H_2*n*+1_^16^O^+^ and **b** C_*n*_H_2*n*+1_^+^ ions (left scale) as well as GC peak areas (right scale) during an *n*-decane/water injection experiment. While C_10_ was held at a constant volume flow of 1 µl/min, different water additions were performed, as highlighted in the colored background fillings

The temporal behavior of peak areas of C_*n*_H_2*n*+1_^16^O^+^ and C_*n*_H_2*n*+1_^+^ ions is presented in Fig. 7. In addition, measured GC peak areas of *n*-decane (squares) are shown for qualitative reference. The water injection periods are marked by different background colors and volume flow annotations. In contrast to the previous experiments, no sharp change in signal levels was observed (either for C_*n*_H_2*n*+1_^16^O^+^ or for C_*n*_H_2*n*+1_^+^), even during the highest water volume flow. Evidently, as envisaged, the ionization conditions seemed to be stabilized by the constant water enrichment of the argon carrier gas, so that additional water did not play a significant role. Hence, using a moistened carrier gas flow and thus keeping the ionization chamber at a high humidity level could allow for the analysis not only of *n*-alkanes but also of *n*-alkane/water mixtures. However, data quality was quite poor in this experiment, as MS peak areas occasionally fluctuated quite heavily. When water was added to the gas stream, the GC data showed some outliers as well. This is probably due to an unsteady evaporation of C_10_ and water, followed by an unequal flow split to the two instruments. Moreover, when comparing MS and GC data development, concentration changes of *n*-decane seem to be better captured by the alkyl fragment signals. The [M−3H+H_2_^16^O]^+^ (C_*n*_H_2*n*+1_^16^O^+^ with *n* = 10, *m/z* 157.1587) signal shows only a slight decrease during water addition and a less dynamic behavior. Hence, although accumulation is less pronounced, there still seems to be a memory effect during [M−3H+H_2_O]^+^ measurement, which averages some dynamics. As the MS method aims for a rapid species analysis to measure system dynamics that are invisible in slow GC separations, this issue has to be further evaluated. Moreover, the suitability of a more complex sample composition (various hydrocarbons and water) as well as a changing syngas matrix still needs to be evaluated. Currently, a method application to online monitoring of a real Fischer–Tropsch product flow is in preparation.

## Conclusion

In this work, we investigated the role of water during APCI(+) online mass spectrometry of *n*-alkanes. By switching between a dry and a wetted argon carrier gas for sample injection, as well as by using ^18^O-labeled water, we revealed that water possesses multiple roles in the hydrocarbon ion formation. First of all, high humidity suppressed the ionization of alkyl (C_*n*_H_2*n*+1_^+^) and alkenyl (C_*n*_H_2*n*−1_^+^) ions. This indicates that proton transfer by H_3_O^+^ was insignificant for C_*n*_H_2*n*+1_^+^ and C_*n*_H_2*n*−1_^+^ formation for volatile *n*-alkanes under the given ionization conditions.

Further, a dual role in the formation of oxygen-containing C_*n*_H_2*n*+1_O^+^ ions was determined. Analyte-specific [M−3H+H_2_O]^+^ formation was presumably based on reactions of primary analyte ions with adsorbed water molecules. During this process, it is assumed that molecules transfer a part of their internal energy to the walls of the ion source. This way, desorbing products did not suffer from major chain cleavages. However, this advantage of [M−3H+H_2_O]^+^ ions was diminished by the fact that gaseous water reacted with adsorbed hydrocarbons (analytes and fragments) from previous sample injections to C_*n*_H_2*n*+1_O^+^ ions, which overlap with signals from the current sample. Evidently, the continuous online sampling led to a severe analyte accumulation in the ion source. This remained rather unnoticed unless water was added.

For a method applied to online analysis of *n*-alkane mixtures, this accumulation or memory effect would cause significant interpretation problems, especially when the water concentration changes over time. A first solution approach, which stabilizes the ionization conditions and minimizes accumulation by keeping the ion source at a continuously high humidity level, was proven valid. This strategy could be promising for analyzing gas flows consisting of *n*-alkanes and water, as for instance in Fischer–Tropsch synthesis. Nevertheless, the reliability of this method in such a technically relevant application still needs to be demonstrated.

### Supplementary Information

Below is the link to the electronic supplementary material.

Supplementary file1 (DOCX 6599 KB)

## Data Availability

All used data can be found in an online repository [27].
