# Supplementary material for: The role of water in APCI-MS online monitoring of gaseous n-alkanes
Source: Anal Bioanal Chem. 2024 Aug 7;416(22):4961–71. doi: 10.1007/s00216-024-05431-5 (PMC11330415; doi:10.1007/s00216-024-05431-5)
Supplement: Supplementary file 1 — Supplementary file1 (DOCX 6599 KB) [file 216_2024_5431_MOESM1_ESM.docx]

**Supporting Information**

**The role of water in APCI-MS online monitoring of gaseous *n*-alkanes**

Jonas Wentrup^a,b^, Thomas Dülcks^c^ , Jorg Thöming^a,b,d*^

^a^ University of Bremen, Faculty of Production Engineering, Chemical Process Engineering,
Leobener Strasse 6, 28359 Bremen Germany

^b^ University of Bremen, Center for Environmental Research and Sustainable Technology,
Postbox 330 440, 28334 Bremen, Germany

^c^ University of Bremen, FB 02, Mass Spectrometry Service Facility, Leobener Str. NW2A, 28359 Bremen, Germany

^d^ University of Bremen, MAPEX Center for Materials and Processes,
Postbox 330 440, 28334 Bremen, Germany

^*^ corresponding author (J. Thöming: [thoeming@uni-bremen.de](mailto:thoeming@uni-bremen.de))

| 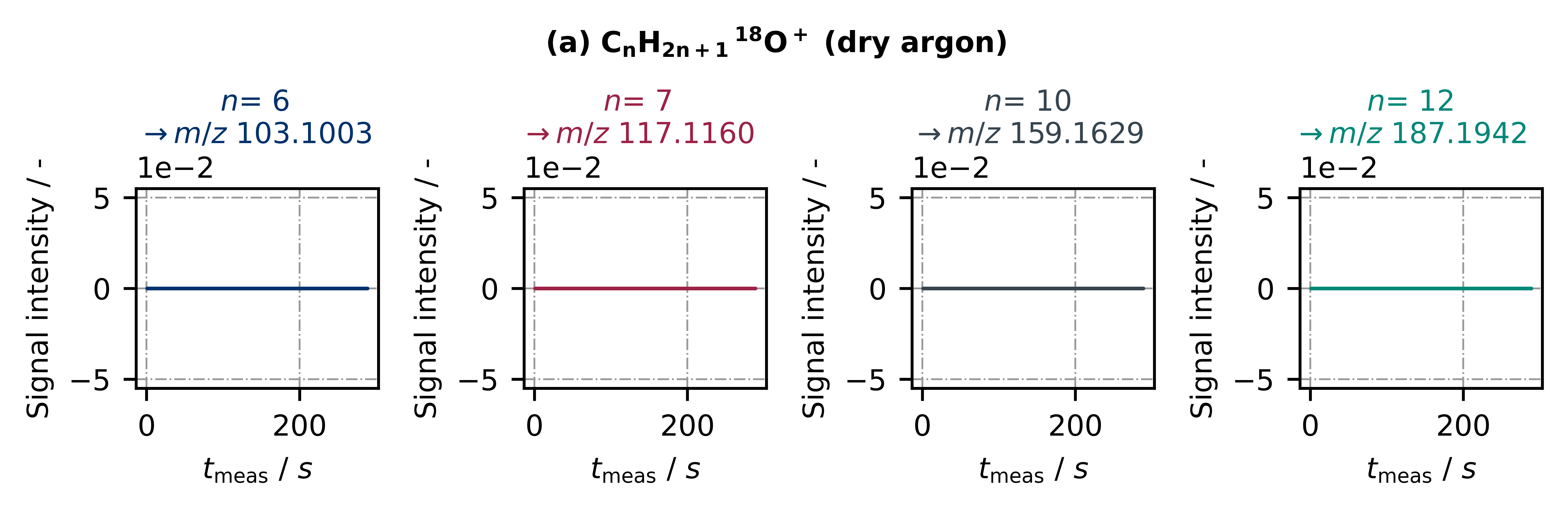 |
| --- |
| 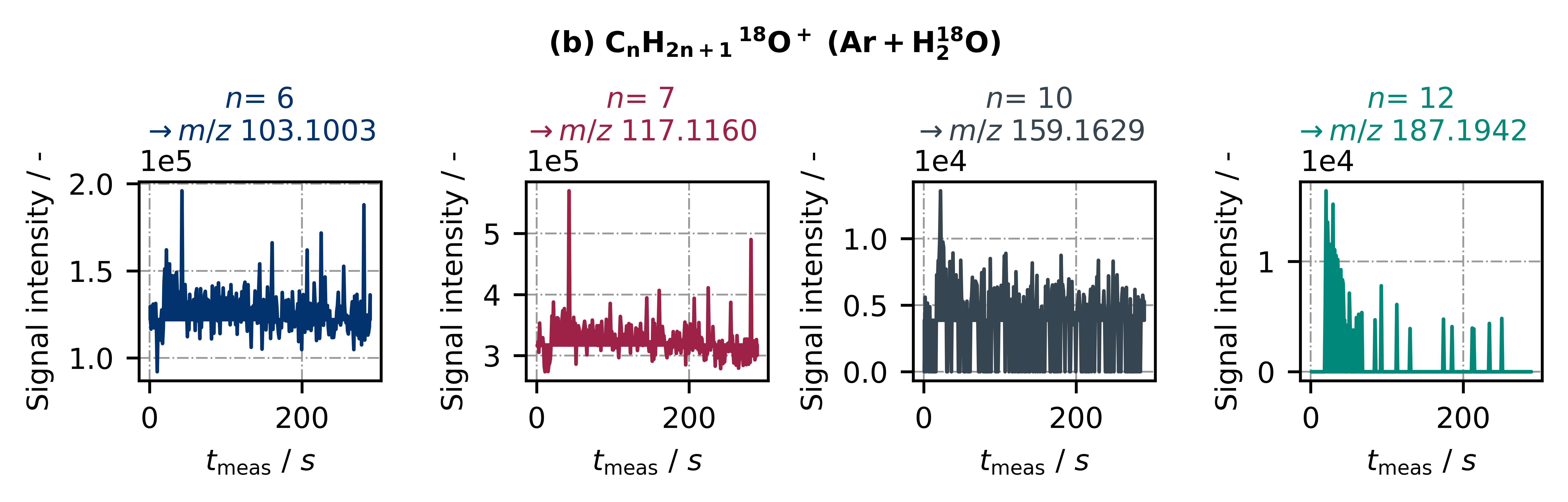 |
| 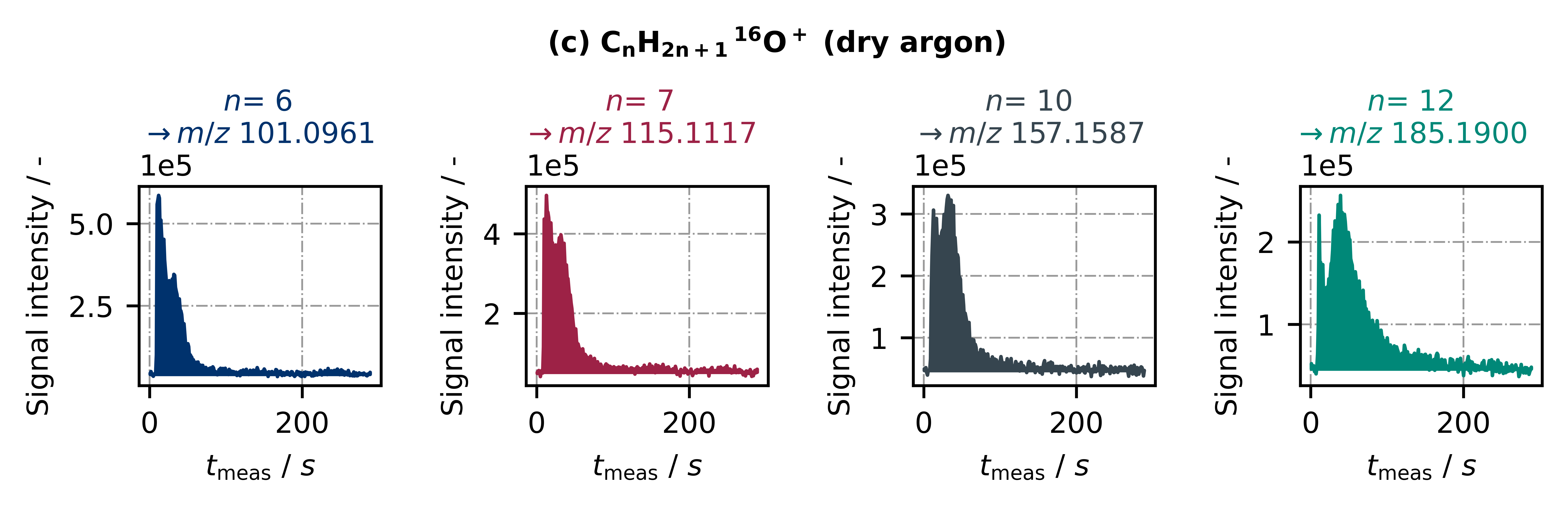 |
| 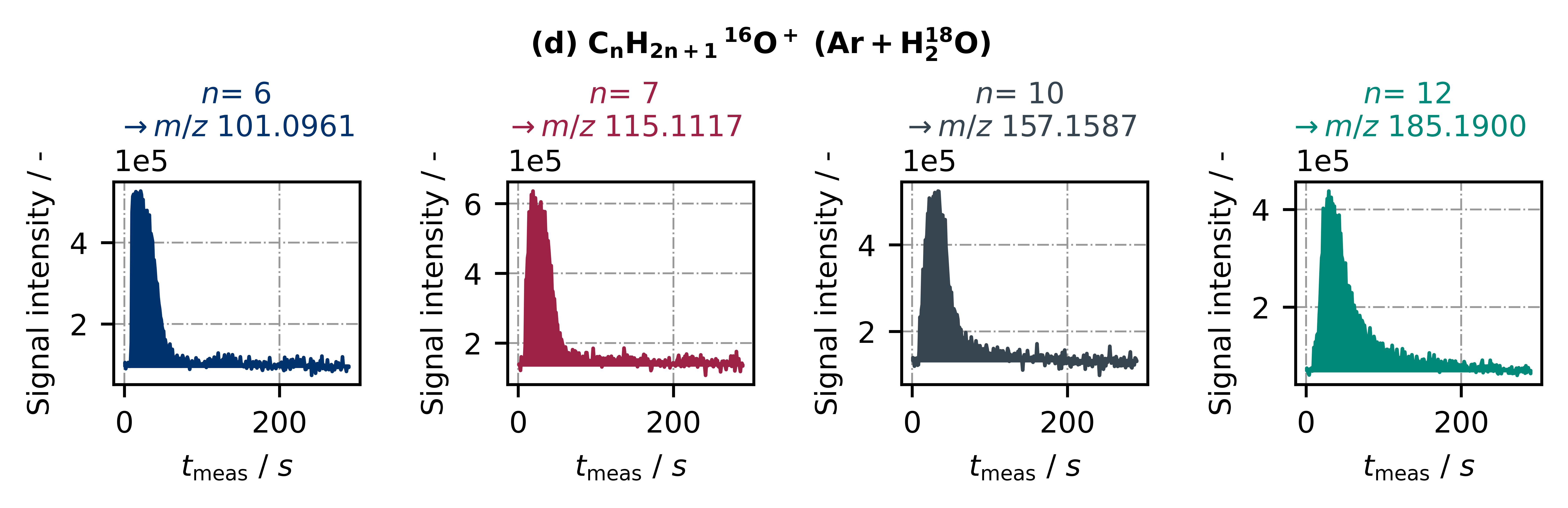 |
| Figure S1: MS ion chromatograms of oxygen-containing C*_n_*H_2_*_n_*_+1_^18^O^+^ (panels (a) and (b)) and C*_n_*H_2_*_n_*_+1_^16^O^+^ (panels (c) and (d)) with the chain lengths *n*=6, 7, 10 and 12, using dry argon carrier gas and H_2_^18^O-enriched argon carrier gas, respectively. The figure refers to Figure 3 from the main manuscript. |
| 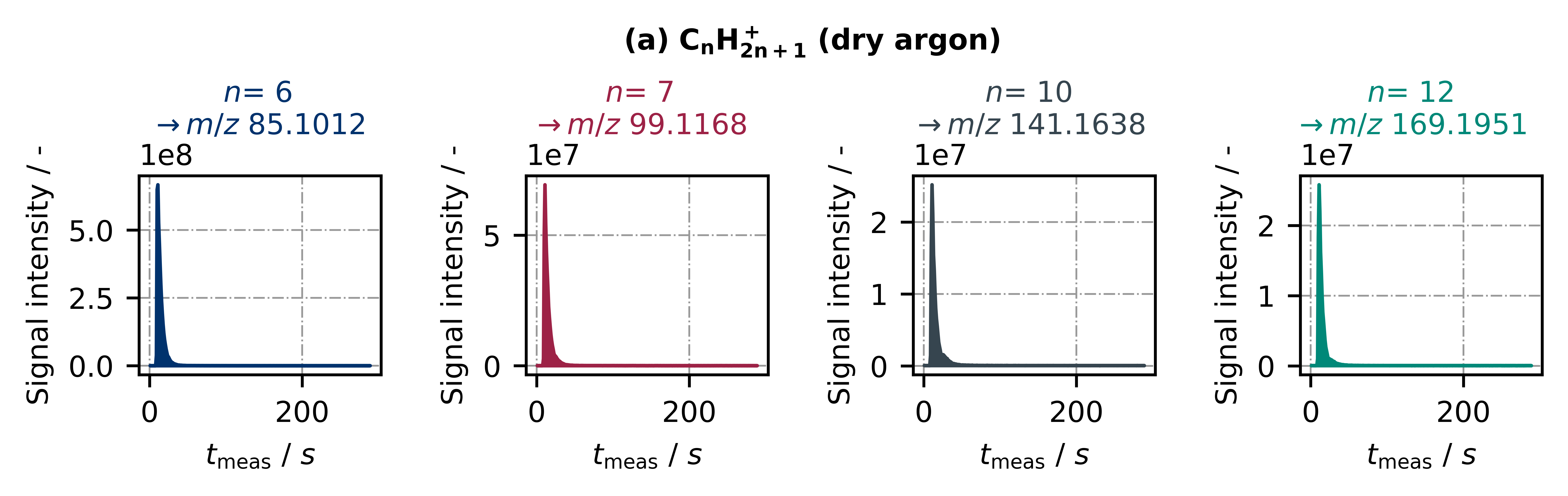 |
| 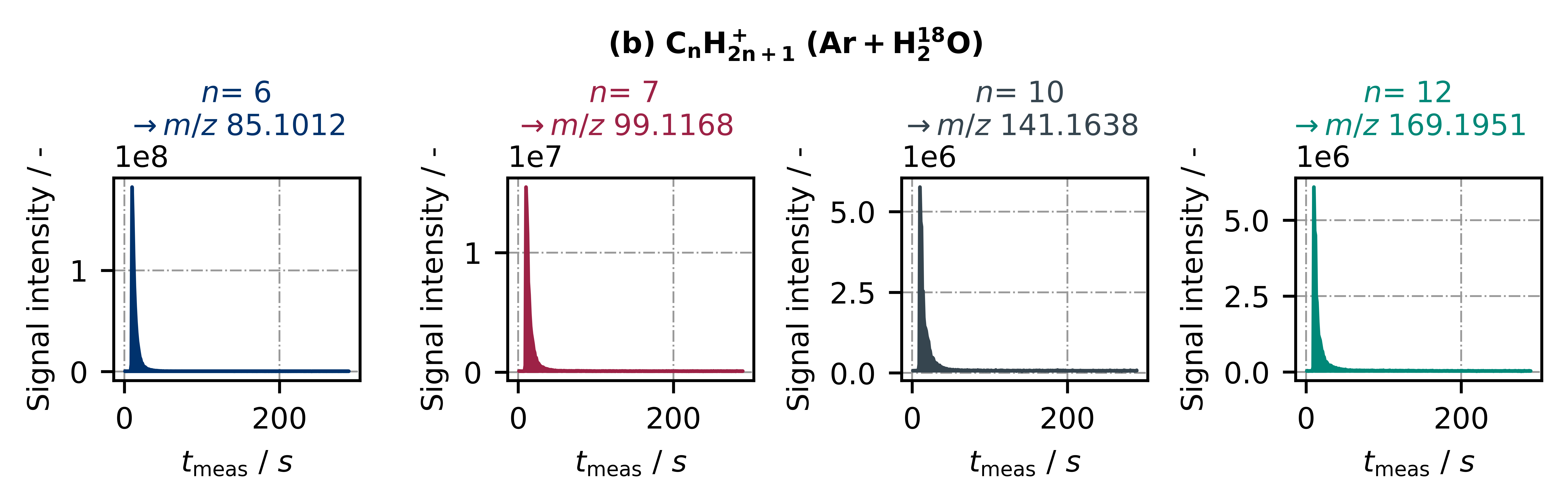 |
| 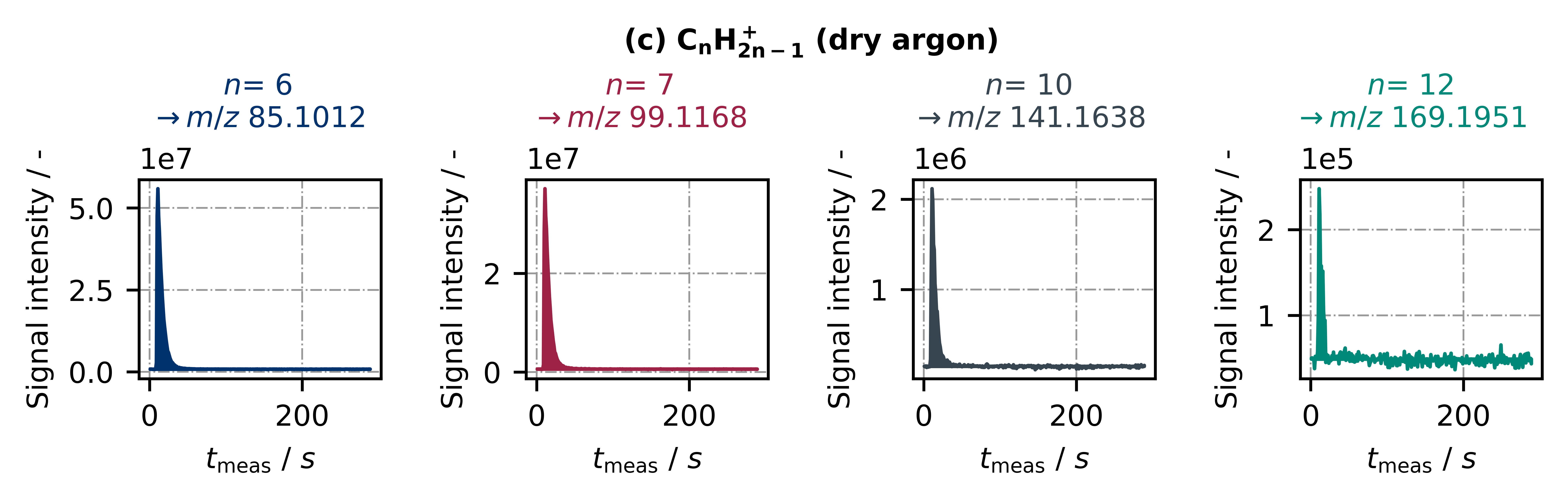 |
| 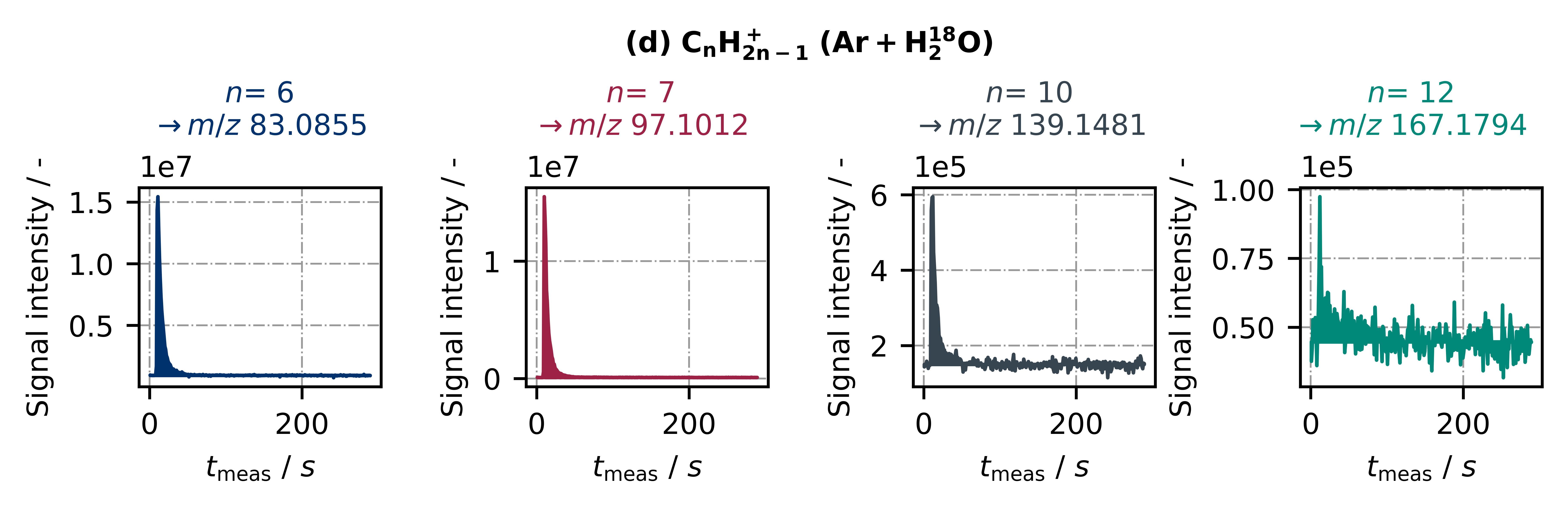 |
| Figure S2: MS ion chromatograms of alkyl (C*_n_*H_2_*_n_*_+1_^+^) ions (panels (a) and (b)) and alkenyl (C*_n_*H_2_*_n-_*_1_^+^) ions (panels (c) and (d)) with the chain lengths *n*=6, 7, 10 and 12, using dry argon carrier gas and H_2_^18^O-enriched argon carrier gas, respectively. The figure refers to Figure 3 from the main manuscript. |

| 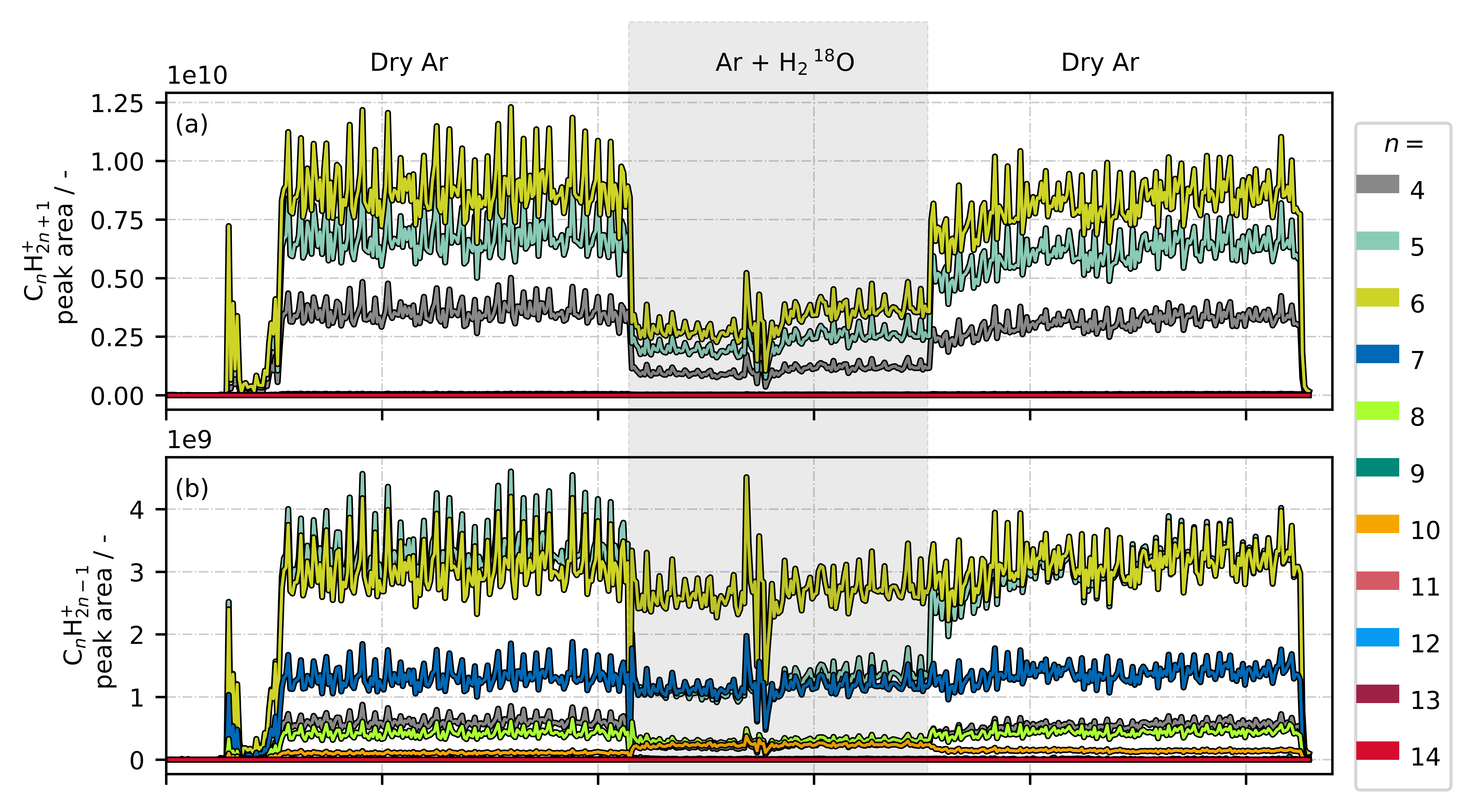 |
| --- |
| Figure S3: Peak areas of the (a) alkyl (C*_n_*H_2_*_n_*_+1_^+^) and (b) alkenyl (C*_n_*H_2_*_n-_*_1_^+^) during a constant *n*-decane injection and a switch between dry (white background) and H_2_^18^O-wetted (shaded background) argon carrier gas. The figure refers to Figure 5 from the main manuscript. |
